# Supplementary figures and images for: Multi-study inference of regulatory networks for more accurate models of gene regulation
Source: PLoS Comput Biol. 2019 Jan 24;15(1):e1006591. doi: 10.1371/journal.pcbi.1006591 (PMC6363223; doi:10.1371/journal.pcbi.1006591)

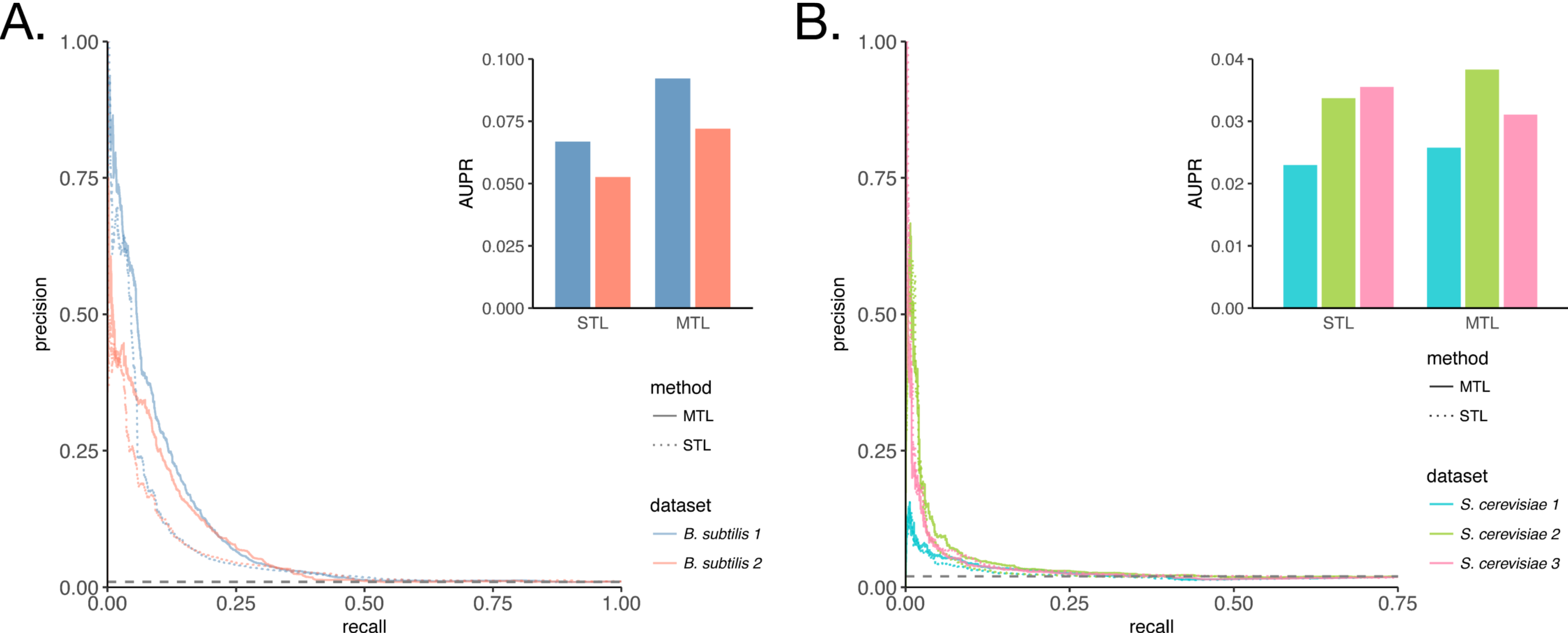

Supplement: S1 Fig — (A) Precision-recall curves assessing accuracy of network models inferred without TF activities for individual B. subtilis datasets against the whole gold-standard set of interactions. Networks Barplot show mean area under precision-recall curve (AUPR) for each method and dataset. (B) Precision-recall curves assessing accuracy of network models inferred without TF activities for individual S. cerevisiae networks, with the difference that priors are derived from chromatin accessibility data. (TIFF) [file pcbi.1006591.s001.tiff]

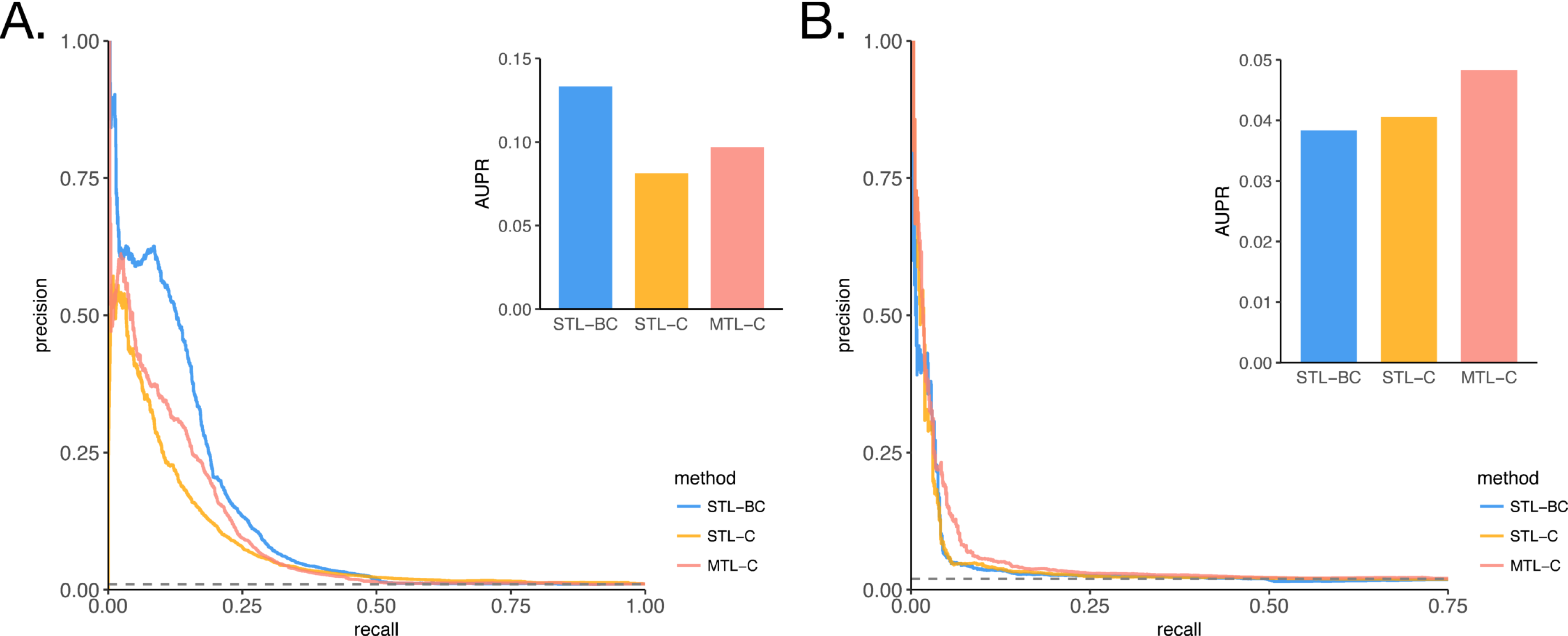

Supplement: S2 Fig — Assessment of accuracy of network models learned using three different data integration strategies, data merging and batch correction (STL-BC), ensemble method combining models learned independently (STL-C), and ensemble method combining models learned jointly (MTL-C). TF expression was used as predictors of gene expression. (A) Precision-recall curves for B. subtilis, again using the whole gold-standard set of interactions. Barplot show mean area under precision-recall curve (AUPR) for each method. (B) Precision-recall curves for S. cerevisiae, with the difference that priors are derived from chromatin accessibility data. (TIFF) [file pcbi.1006591.s002.tiff]
